# PIK3R2 immunostaining status predicts prognosis in patients with newly diagnosed glioblastoma treated with an autologous tumor vaccine

**DOI:** 10.1007/s11060-025-05102-0

**Published:** 2025-06-12

**Authors:** Kazuki Akutagawa, Shunichiro Miki, Erika Yamada, Noriaki Sakamoto, Tsubasa Miyazaki, Narushi Sugii, Alexander Zaboronok, Masahide Matsuda, Eiichi Ishikawa

**Affiliations:** 1 Department of Neurosurgery, Institute of Medicine, University of Tsukuba, 1-1-1 Tennodai, Tsukuba, Ibaraki Prefecture 305-8575 Japan; 2 Department of Diagnostic Pathology, Institute of Medicine, University of Tsukuba, 1-1-1 Tennodai, Tsukuba, Ibaraki 305-8575 Japan; 3 Cell-Medicine, Inc, Sengen 2-1-6, Tsukuba Science City, Ibaraki 305-0047 Japan

**Keywords:** Immunotherapy, Tumor vaccine, Prognosis, PIK3R2, PI3K pathway, Glioblastoma

## Abstract

**Background:**

Glioblastoma (GBM) is the most common and aggressive primary brain tumor in adults, characterized by high invasiveness and a poor prognosis, with limited treatment options. Our previous study on fractionated radiotherapy, temozolomide, and an autologous formalin-fixed tumor vaccine (AFTV) for newly diagnosed grade 4 astrocytic tumors demonstrated that complete tumor resection and p53 negativity on immunohistochemistry were associated with favorable outcomes. PIK3R2, a key component of the PI3K–Akt signaling pathway, may modulate the host immune response to tumor antigens and influence the efficacy of immunotherapy. In this study, we further investigated whether PIK3R2, a candidate gene identified through gene panel sequencing as potentially associated with prognosis following AFTV treatment, influences patient outcomes after AFTV therapy.

**Methods:**

We analyzed 58 patients with newly diagnosed IDH wildtype GBM or IDH mutant grade 4 astrocytoma (Astro). Among them, 29 received standard treatment combined with AFTV (AFTV group), while 29 underwent standard treatment alone (control group). Immunostaining for PIK3R2 and p53 was performed, and patient characteristics, including age, sex, Karnofsky Performance Status at the time of surgery, and overall survival (OS), were evaluated. PIK3R2 expression levels were classified using a 34% cutoff value.

**Results:**

In the AFTV group, survival analysis based on PIK3R2 status (positive/negative) revealed an increased survival in the PIK3R2-negative group when comparing AFTV and control groups (*p* = 0.075 in GBM/Astro cases and *p* = 0.030 in GBM cases). When stratifying patients into four subgroups based on p53 and PIK3R2 status (p53-negative/PIK3R2-negative, p53-positive/PIK3R2-positive, p53-negative/PIK3R2-positive, and p53-positive/PIK3R2-negative), a significant improvement in OS was observed in the p53-negative/PIK3R2-negative group both in GBM/Astro cases and GBM cases. PD-1 demonstrated the strongest correlation with PIK3R2 in the regression analysis.

**Conclusion:**

Negative immunostaining for PIK3R2 as well as negative p53 revealed an increased survival in patients receiving AFTV therapy for GBM. In patients receiving AFTV, these immunostaining results may serve as a predictor of treatment efficacy and overall survival.

**Supplementary Information:**

The online version contains supplementary material available at 10.1007/s11060-025-05102-0.

## Introduction

Glioblastoma (GBM) is the most aggressive and prognostically unfavorable primary brain tumor in adults, with limited treatment options. Autologous formalin-fixed tumor vaccine (AFTV) is a novel therapeutic approach developed through a collaborative research project between our department and the RIKEN Institute (Japan) [1, 2]. This treatment is based on a unique concept utilizing non-cultured, non-synthesized autologous tumor-associated antigens [1]. Compared with other immune checkpoint inhibitors or peptide vaccines, AFTV may provide broader antigen coverage and a more favorable safety profile, as previously reported [2, 3]. Phase I/IIa and IIb trials conducted by our institution and collaborating centers demonstrated the safety of AFTV therapy when administered alongside standard treatment for patients with newly diagnosed GBM [2, 3]. Based on these findings, a phase III investigator-led clinical trial was initiated in 2020 to evaluate its efficacy (jRCT2031200153). Our previous studies indicated that a subset of GBM patients who underwent maximal surgical resection achieved long-term survival when AFTV was administered intradermally in the upper arm in combination with standard postoperative chemoradiotherapy and maintenance chemotherapy. However, some patients experienced early recurrence with no improvement in prognosis. Therefore, identifying predictive factors for therapeutic response is essential for optimizing treatment strategies for newly diagnosed GBM [2, 3]. To date, however, no reliable predictive biomarkers have been established for GBM immunotherapy. Subsequent research revealed that p53 negativity on immunostaining was associated with a favorable prognosis in patients receiving AFTV therapy [4]. In our previous study, next-generation sequencing panel tests (Qiagen QIAseq Targeted DNA Panels)_of 27 tumor samples (13 in the AFTV group and 14 in the control group) [4] identified mutations in PIK3R2, also known as p85β, a Class I regulatory subunit of phosphatidylinositol 3-kinase (PI3K), as a potential prognostic factor (Supplementary Table 1). In this preliminary study, mutant PIK3R2 (p85β) was associated with a favorable prognosis in patients receiving AFTV therapy, but not in patients with standard therapy. Additionally, GlioVis analysis using HG-U133A gene expression data indicated that PIK3R2 gene expression was not associated with prognosis in patients undergoing standard treatment (Supplementary Fig. 1; see figure legend for GlioVis Explore settings).

PIK3R2 is a key component of the PI3K–Akt signaling pathway, which plays a central role not only in tumor proliferation but also in immune regulation, including T-cell exhaustion, dendritic cell function, and tumor metabolism [5]. These biological features suggest that PIK3R2 may modulate the host immune response to tumor antigens and influence the efficacy of immunotherapy such as AFTV. Although no studies to date have demonstrated a direct association between PIK3R2 mutations and immunohistochemical (IHC) staining patterns, certain mutations may result in N-terminal or C-terminal truncations of the protein [6]. This raised the possibility that such mutations could lead to negative IHC staining depending on the antibody’s epitope recognition. Based on this hypothesis, we aimed to evaluate protein expression by immunohistochemistry. In this study, we performed an immunohistochemical analysis of PIK3R2 and assessed its potential as a predictive biomarker in combination with p53 staining for AFTV-treated patients.

## Materials and methods

Patients diagnosed with and treated for newly diagnosed GBM according to the 2016 World Health Organization (WHO) classification (IDH wildtype GBM or IDH mutant astrocytoma grade 4 on the 2021 WHO classification) at our hospital between May 2007 and December 2018 were included in this retrospective study, as previously described [4]. In this manuscript, the term “GBM” refers specifically to IDH-wildtype glioblastoma, in accordance with the WHO 2021 classification. The term “GBM/Astro” refers to the broader cohort that includes both IDH-wildtype GBM and IDH-mutant grade 4 astrocytoma, as defined by the WHO 2016. IDH status was primarily determined by immunohistochemistry using an anti-IDH1 R132H antibody and was further confirmed by Sanger sequencing or next-generation sequencing in patients under 55 years of age, as described in our previous report [4].

A total of 29 patients who received standard treatment combined with AFTV therapy (AFTV group) and 29 propensity score-matched patients with newly diagnosed GBM received standard treatment alone from the same period (control group) were selected [4]. Consequently, the control group in this study exhibited a relatively favorable prognosis, with a median overall survival (mOS) of 31.1 months, which is longer than typically observed in standard control groups. Newly obtained PIK3R2 protein expression data, evaluated by IHC staining, were analyzed in conjunction with the clinical dataset used in our previous study [4]. This dataset included patient characteristics such as age, sex, Karnofsky Performance Status (KPS) at the time of surgery, overall survival (OS), and p53 positivity determined using the anti-p53 antibody (DO-7, Agilent Technologies, Santa Clara, CA, USA). Additionally, scores for various immune markers, including CD3 (2GV6, Ventana), CD8 (C8/144B, Dako), programmed cell death protein 1 (PD-1, NAT105, Cell Marque), programmed cell death ligand 1 (PD-L1, clone SP263, Roche), and CD163 (10D6, Novocastra), were evaluated using IHC staining. For p53, positivity was defined as nuclear staining in ≥ 10% of tumor cells, using the DO-7 monoclonal antibody, which is a reliable surrogate marker of missense TP53 mutations [7]. Scoring for immune markers was performed using marker-specific methods. For PD-1, positive immune cells were scored as follows: 1 point (0–4 cells per high-power field at ×400 magnification), 2 points (5–8 cells), 3 points (9–12 cells), and 4 points (> 13 cells). PD-L1 was scored on a scale of 2 to 8 based on the percentage of positive tumor cells. Scoring for other markers was based on the average number of positive cells in six high-power fields, as described in our previous report [4]. This study was conducted with the approval of the University of Tsukuba Ethics Committee (approval number: R01-165).

### Immunohistochemistry for PIK3R2

**Fig. 1:**
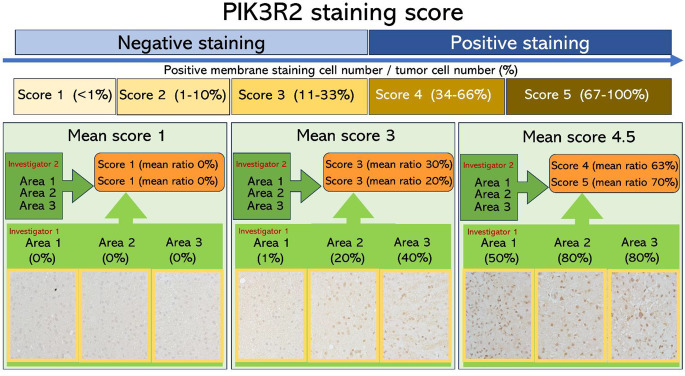
PIK3R2 staining score. PIK3R2 protein expression was evaluated based on the percentage of tumor cell membranes showing positive staining in three randomly selected high-power fields. Staining scores were assigned as follows: score 0 (no immunoreactivity), score 1 (< 1%), score 2 (1–10%), score 3 (11–33%), score 4 (34–66%), and score 5 (67–100%). Two investigators independently and blindly assessed the stained sections. A mean score of ≥ 4 was classified as PIK3R2-positive, while a score of < 4 was classified as PIK3R2-negative

IHC staining was performed using an anti-PIK3R2 antibody (AB180967, Abcam, Cambridge, UK). PIK3R2 protein expression was evaluated by calculating the mean percentage of positively stained tumor cell membranes across three randomly selected high-power fields. Staining intensity was scored based on the percentage of membrane-positive tumor cell as follows: score 0 (no immunoreactivity), score 1 (< 1%), score 2 (1–10%), score 3 (11–33%), score 4 (34–66%), and score 5 (67–100%) (Fig. 1). Two investigators independently and blindly assessed the staining, and the mean values were used for analysis. For classification purposes, PIK3R2 protein expression was considered “positive” when the mean score was ≥ 4 (≥ 34% membrane positivity), and “negative” when the mean score was < 4 (< 34%). This cutoff was based on a previous breast cancer study that applied a similar scoring system for PIK3R1 staining [8], and it provided the greatest separation in survival curves in our preliminary analyses.

### Statistical analysis

All results are presented as the number of cases (%) or median values (range). Statistical analyses were performed using IBM SPSS Statistics version 27.0 (IBM Corporation, Armonk, NY, USA). Median OS, or mOS, defined as the time from diagnosis to death or the last follow-up, was estimated using the Kaplan-Meier method. Survival differences between the AFTV and control groups were assessed using the log-rank test, with statistical significance set at *p* < 0.05.

The Mann–Whitney U test (MWU) was used to compare continuous variables and ordinal scales between the AFTV and control groups, while Fisher’s exact test was applied for categorical (nominal) variables. Correlation analysis was performed using a linear regression model.

## Result

The overall patient characteristics were as follows: the mean age was 57 years, with 67.2% of patients aged 55 or older, and 21 patients (36.2%) were female. The median Karnofsky Performance Status (KPS) at the time of surgery was 80, and the mOS was 29.8 months. In the AFTV group, the mean age was 59.1 years, and 14 patients (48.3%) were female. The median KPS at the time of surgery was 80, and the mOS was 32.6 months. There were more female patients in the AFTV group compared to the control group (*p* = 0.1), but this was not statistically significant (Table 1). Regarding IHC staining results, p53-negative status was observed in 13 cases (44.8%), and PIK3R2-negative status in 10 cases (34.5%) within the AFTV group. No significant differences in these markers were detected between the AFTV and control groups (Fisher’s exact test, *p* = 0.839). Additionally, there was no significant correlation between p53 status and PIK3R2 scores (median score = 4 in both p53-negative and p53-positive cases; Mann–Whitney U test, *p* = 0.369).

**Table 1:** Patient characteristics

Characteristic	AFTV group (*N* = 29)	Control group (*N* = 29)	Total (*N* = 58)	*P*-value
Age (years, mean [range])	59.1 (39–74)	55.3 (14–85)	57.2 (14–85)	> 0.1
Sex (female, n [%])	14 (48.3)	7 (24.1)	21 (36.2)	0.100
Preoperative KPS (median [range])	80 (50–100)	90 (50–100)	80 (50–100)	> 0.1
Overall survival (months, median [range])	32.6 (6.9-118.2)	31.1 (2.8–82.2)	31.1 (2.8-118.2)	> 0.1
IDH status				> 0.1
Wildtype	24 (82.8)	23 (79.3)	47 (81.0)	
Mutant	5 (17.2)	4 (13.8)	9 (15.5)	
Not determined	0 (0.0)	2 (6.9)	2 (3.4)	
p53 status, n (%)				> 0.1
Negative (< 10%)	13 (44.8)	13 (44.8)	26 (44.8)	
Positive (≥ 10%)	15 (51.8)	16 (55.2)	31 (53.5)	
Not available (N/A)	1 (3.4)	0 (0.0)	1 (1.7)	
PIK3R2 status, n (%)				> 0.1
Negative (< 34%)	10 (34.5)	13 (44.8)	23 (39.7)	
Positive (≥ 34%)	17 (58.6)	15 (51.8)	32 (55.1)	
Not available (N/A)	2 (6.9)	1 (3.4)	3 (5.2)	

**Fig. 2:**
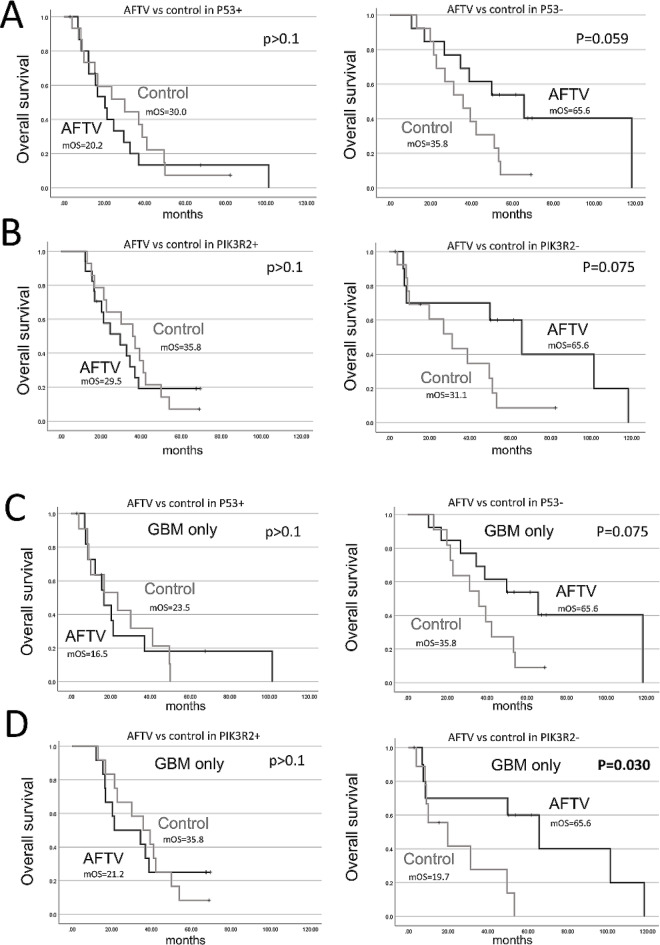
Survival analyses in the autologous formalin-fixed tumor vaccine (AFTV) group and control group, adjusted for prognosis, across two subgroups: (**A**) p53 status (positive/negative) in cases of glioblastoma (GBM) and grade 4 astrocytoma (Astro) (GBM/Astro), (**B**) PIK3R2 status (positive/negative) in GBM/Astro cases, (**C**) p53 status in GBM cases, and (**D**) PIK3R2 status in GBM cases

Survival analyses were performed for the AFTV and control groups by dividing patients into four subgroups based on p53 (positive/negative) and PIK3R2 (positive/negative) status. The results for p53 status are shown in Fig. 2A and Supplementary Fig. 2A, and those for PIK3R2 status are shown in Fig. 2B and Supplementary Fig. 2B. In the AFTV group, the mOS was 20.2 months for the p53-positive subgroup, 29.5 months for the PIK3R2-positive subgroup, and 65.6 months for both p53-negative and PIK3R2-negative subgroups. In the control group, survival times were similar across all subgroups: 30.0 months for the p53-positive subgroup, 35.8 months for the PIK3R2-positive subgroup, 35.8 months for the p53-negative subgroup, and 31.1 months for the PIK3R2-negative subgroup. Although survival tended to be longer in the PIK3R2-negative subgroup of the AFTV group compared to the corresponding control group, this difference did not reach statistical significance (log-rank test, *p* = 0.075). Among patients with GBM, the p53-negative subgroup in the AFTV group showed a trend toward improved survival (65.6 months mOS) compared to the same subgroup in the control group (35.8 months). Notably, the PIK3R2-negative subgroup of the AFTV group demonstrated a significantly longer mOS (65.6 months) compared to the corresponding subgroup in the control group (19.7 months; log-rank test, *p* = 0.030, Fig. 2C and D).

**Fig. 3:**
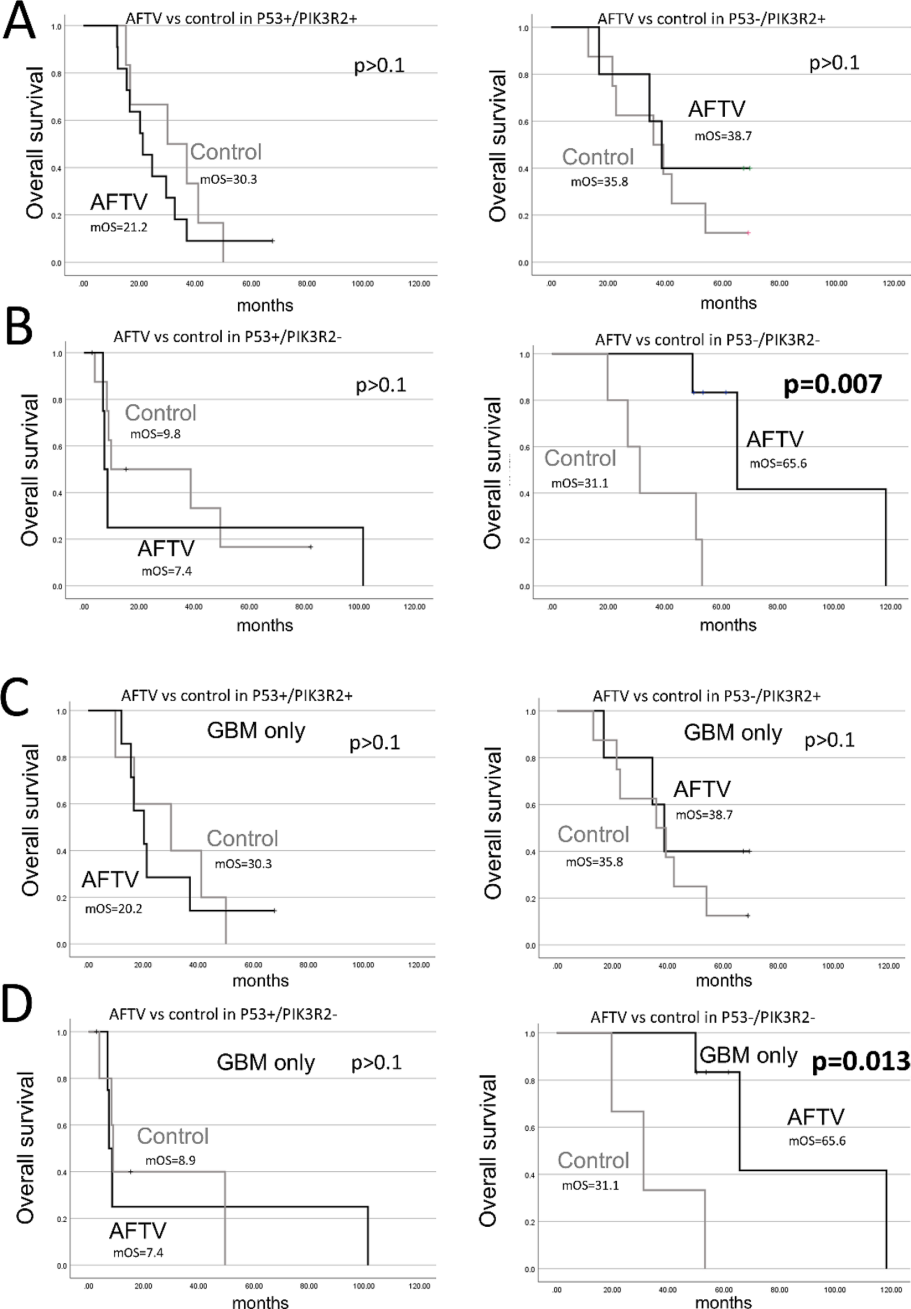
Comparison of mOS between the AFTV and control groups, stratified by p53 status and PIK3R2 status in GBM/Astro cases (**A** and **B**) and GBM cases (**C** and **D**)

When comparing the AFTV and control groups across four combined subgroups (p53-negative/PIK3R2-negative, p53-positive/PIK3R2-positive, p53-negative/PIK3R2-positive, and p53-positive/PIK3R2-negative), a significant increase in mOS was observed only in the p53-negative/PIK3R2-negative subgroup (65.6 months in the AFTV group vs. 31.1 months in the control group, *p* = 0.007, log-rank test) (Fig. 3A and B, and Supplementary Fig. 3). No significant differences were observed in the other three subgroups (*p* > 0.05, log-rank test). Similar results were observed when the analysis was limited to GBM cases (Fig. 3C and D).

**Fig. 4:**
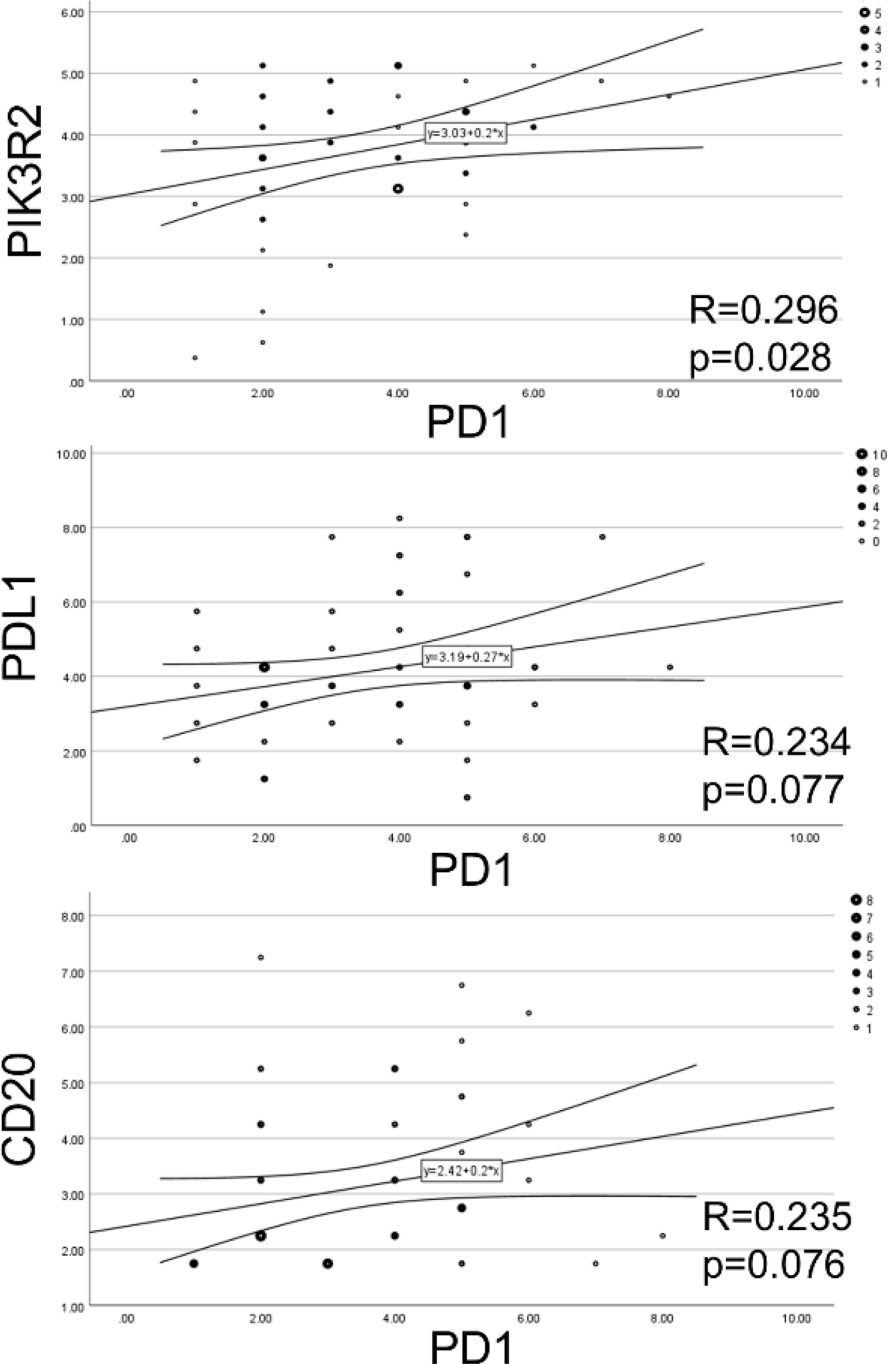
Association between immune marker scores in GBM/Astro cases: (**A**) PIK3R2 and PD-1, (**B**) PD-L1 and PD-1, and (**C**) CD20 and PD-1, analyzed using the Pearson correlation coefficient

**Fig. 5:**
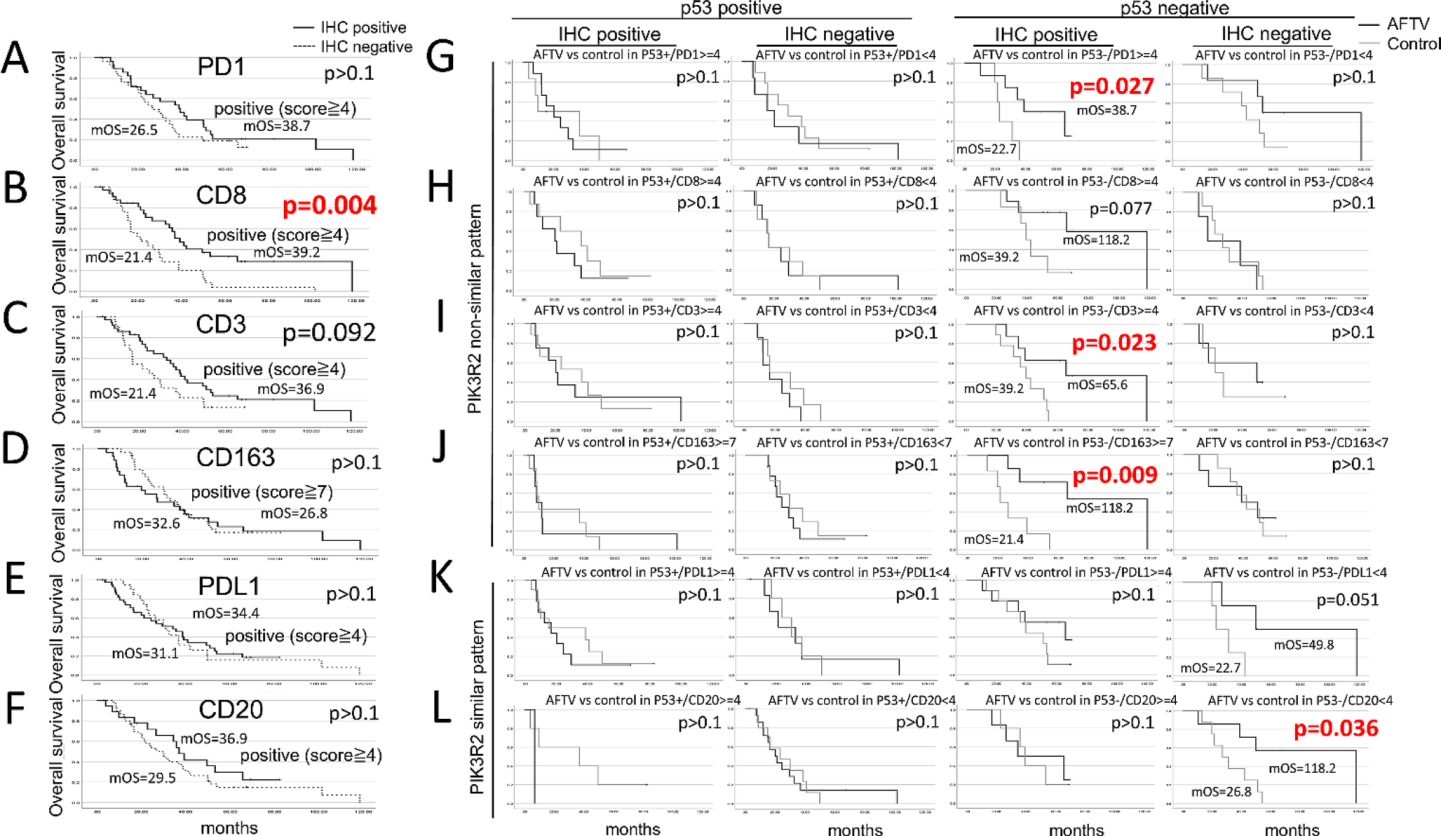
Survival analyses based on PD-1, CD8, CD3, CD163, PD-L1, and CD20 status in the AFTV and control groups in GBM/Astro cases. (**A-F**) Survival analyses in the entire patient cohort, stratified by PD-1, CD8, CD3, CD163, PD-L1, and CD20 status (positive/negative). (**G-L**) The AFTV and control groups were each subdivided into four groups based on p53 status and the status of each molecule, and survival outcomes were compared

Next, we examined the correlation between immune cell staining scores and PIK3R2 scores, as shown in Table 2; Fig. 4. Among the immune markers analyzed, PD-1 demonstrated the strongest correlation with PIK3R2 in the regression analysis (Table 2, upper panel). Additionally, PD-1 scores showed a tendency to correlate with CD3, PD-L1, and CD20 scores (Table 2, lower panel). Survival analyses in the entire cohort of 56 patients, based on the IHC status (positive/negative) of PD-1, CD8, CD3, CD163, PD-L1, and CD20, revealed no statistically significant differences (*p* > 0.05, log-rank test), except for CD8 status (Fig. 5A-F). Further subgroup analyses were performed within the AFTV and control groups, based on p53 status combined with IHC status of the above immune markers (Fig. 5G-L, suppl. Table 2). A significant increase or trend toward an increase in OS was observed in the AFTV group for the following subgroups: p53-negative/PD-1-positive, p53-negative/CD8-positive, p53-negative/CD3-positive, p53-negative/CD163-positive, p53-negative/PD-L1-negative, and p53-negative/CD20-negative. No significant survival differences were observed in the other four subgroups (*p* > 0.1, log-rank test).

**Table 2:** Association between PIK3R2, PD-1, and immune cell markers

Immune cell marker		Linear regression model			*R*
	Immune cell marker	*B* value	Standard error	*p*-value	
PIK3R2	CD3	-0.028	0.103	> 0.1	0.037
	CD8	0.013	0.088	> 0.1	0.021
	PD1	0.203	0.090	0.028	0.296
	PDL1	0.029	0.081	> 0.1	0.047
	CD20	-0.027	0.110	> 0.1	0.033
	CD163	-0.111	0.101	> 0.1	0.149
PD1	CD3	0.244	0.137	0.081	0.231
	CD8	0.119	0.123	> 0.1	0.128
	PDL1	0.208	0.114	0.077	0.234
	CD20	0.274	0.151	0.076	0.235
	CD163	0.139	0.146	> 0.1	0.126

## Discussion

In GBM, following standard treatment, key prognostic factors include age, preoperative performance status, tumor location, preoperative imaging characteristics, and the extent of resection [9, 10]. O6-Methylguanine-DNA methyltransferase (MGMT) promoter methylation is a well-established prognostic biomarker for GBM, regardless of treatment modality [11], and is also associated with a favorable response to temozolomide (TMZ). In patients with MGMT promoter methylation, combination therapy significantly improves progression-free survival (PFS) and OS compared to either treatment alone [12]. However, in our previous study [4], MGMT promoter methylation status did not influence prognosis in AFTV-treated patients, highlighting the need for novel prognostic markers in this setting. In this study, we analyzed patient characteristics and survival outcomes in the AFTV and control groups. IHC staining revealed a significant difference in PIK3R2-negative status between the two groups of GBM cases. Additionally, survival analysis demonstrated a significant increase in mOS in the AFTV group compared to the control group, particularly in patients with p53-negative/PIK3R2-negative status, while no significant survival differences were observed in the other subgroups. Furthermore, PIK3R2 staining scores were found to correlate directly with PD-1 staining scores and indirectly with PD-L1 staining scores. Survival analysis based on PD-L1 status showed a significant survival advantage in the AFTV group for patients with p53-negative/PD-L1-negative status. These findings suggest that AFTV treatment may provide a survival benefit, particularly in specific molecular subgroups, underscoring the potential role of PIK3R2 and immune-related markers in predicting treatment response. PIK3R2 encodes a Class I regulatory subunit of PI3K, an enzyme that phosphorylates phosphatidylinositol-4,5-bisphosphate (PIP2) to generate phosphatidylinositol-3,4,5-trisphosphate (PIP3), a key component in intracellular signaling [13]. The PI3K/AKT pathway plays a critical role in cell survival and apoptosis (programmed cell death) inhibition [14]. Additionally, PIK3R2 facilitates cell cycle progression and proliferation, functioning as a potential tumor-promoting factor [15]. Furthermore, the pathway is a well-established oncogenic mechanism in GBM and is also involved in immune response regulation [16]. The altered RTK/RAS/PI3K/AKT pathway has been associated with tumor mutation burden and predictive markers for immunotherapy, including tumor-infiltrating CD8-positive T cells [16]. PIK3R2 is involved in immune cell activation, differentiation, and cytokine production through this pathway [17].

The previous study on *PIK3R1/2* gene expression in various malignant tumors has shown that *PIK3R2* is highly expressed in most tumors and is associated with poor prognosis in certain types of malignancies [18]. However, in GBM, the prognostic significance of PIK3R2 gene and protein expression remains poorly understood. To date, only one study has reported that overexpression of lncRNA XLOC013218 markedly increases TMZ resistance, promotes proliferation, and inhibits apoptosis through PIK3R2-mediated activation of the PI3K/AKT signaling pathway [18]. Additionally, TP53 may regulate cell survival signaling by suppressing the PI3K/Akt/mTOR pathway via *IGF-BP3*, *PTEN*, and *TSC2* signaling [14, 19]. Furthermore, mutations activating the PI3K/Akt/mTOR pathway are known to increase glycolysis in tumor cells and impair effector T-cell function by depleting environmental glucose [20, 21].

PIK3R2 protein expression status did not influence prognosis in the standard treatment group in our study. This suggests that the improved survival observed in the p53-negative/PIK3R2-negative subgroup of the AFTV group may be attributed to the effects of AFTV treatment. Tumors with functional p53 and inactivation of the PI3K/Akt/mTOR pathway generally exhibit lower metabolic activity, slower growth, and a reduced likelihood of generating immune escape clones. These characteristics suggest that p53-negative/PIK3R2-negative tumors in this study may represent optimal targets for immunotherapy.

In this study, IHC analysis showed a significant correlation between PIK3R2 and PD-1 staining scores, as determined by linear regression analysis. Previous studies have reported that PI3K signaling induces the formation of myeloid-derived suppressor cells (MDSCs) and M2 macrophages (M2Mφ), leading to an immunosuppressive tumor environment [22, 23]. Among these immune subsets, *PIK3R2* gene expression has been specifically associated with dendritic cell infiltration in GBM [13]. High *PIK3R2* gene expression sustains PI3K pathway activation, promoting T-cell exhaustion and contributing to an immunosuppressive tumor microenvironment [22, 24]. Additionally, PD-1 functions as an immune checkpoint not only on T lymphocytes but also on B lymphocytes, natural killer cells, and macrophages [25]. Based on these findings, we speculate that high *PIK3R2* gene expression plays a role in both B-cell induction and B-cell exhaustion. Furthermore, we hypothesize that in p53-negative gliomas, *PIK3R2* inactivation may promote an enhanced immune response and suppress the PD-1/PD-L1 pathway within the tumor microenvironment. This could facilitate effective immunotherapy, preventing B- and T-cell exhaustion even after treatment. However, as the prognostic impact of PIK3R2 expression cannot be solely explained by the PD-1/PD-L1 pathway, further studies are needed to elucidate the underlying mechanisms.

Our study also suggests the potential benefit of additional PI3K pathway inhibition, particularly in PIK3R2-positive tumors. PI3Kγ inhibitors have been shown to enhance therapeutic efficacy when combined with immune checkpoint inhibitors [26, 27]. Although buparlisib, a Class I PI3K inhibitor, demonstrated limited efficacy as monotherapy in recurrent GBM, it holds promise as a combination agent with immunotherapy [28]. A more recent study reported that PIK3R1 is uniquely overexpressed in IDH1-mutant grade 4 astrocytoma, indicating that combined targeting of the PI3K/AKT/mTOR pathway and the immune system may be necessary for effective treatment in this subtype [29]. Therefore, clinical trials evaluating the efficacy of PI3K inhibitors in combination with immunotherapy in patients with GBM and grade 4 astrocytoma, particularly those less likely to respond to immunotherapy alone, are warranted.

This study has several limitations. First, its retrospective, single-center design and relatively small sample size may limit the generalizability of the findings. The small cohort size could also reduce statistical power, particularly in subgroup analyses, and precluded the use of multivariate analysis due to the limited number of events. The inclusion of both IDH wild-type GBM and IDH mutant grade 4 astrocytoma, along with age and sex imbalances in the control and AFTV groups, may have introduced heterogeneity and influenced the interpretation of survival outcomes, although a sub-analysis restricted to IDH wild-type GBM yielded similar trends. Additionally, while statistical analyses were performed on IHC data, no external validation cohort was included. The relationship between *PIK3R2* gene mutation, gene expression, and protein expression was not assessed in this study. Further preclinical studies and prospective multicenter trials are warranted to validate and extend our findings.

In conclusion, negative immunostaining for PIK3R2 may serve as a predictive biomarker for improved outcomes in patients receiving AFTV therapy for GBM. Furthermore, p53-negative/PIK3R2-negative status was shown to be a strong favorable prognostic factor for AFTV treatment. In patients receiving AFTV, the combination of p53 and PIK3R2 immunostaining results may serve as a predictor of treatment efficacy and patient outcome.

## Electronic supplementary material

Below is the link to the electronic supplementary material.

Supplementary Material 1

## Data Availability

Raw data were generated at the Department of Neurosurgery, University of Tsukuba. Derived data supporting the results of this study are available from the corresponding author (E.I.) on request.

## Abbreviations

AFTV: Autologous formalin-fixed tumor vaccine
APP: Antigen processing and presentation
IHC: Immunohistochemical
KPS: Karnofsky Performance Status
OS: Overall survival
mOS: Median overall survival
MWU: Mann–Whitney U test
PD-1: Programmed cell death protein 1
PD-L1: Programmed cell death ligand 1
PI3K: Phosphatidylinositol 3-kinase
PS: Performance status
SNPs: Single nucleotide polymorphisms
