# Correction to: PIK3R2 immunostaining status predicts prognosis in patients with newly diagnosed glioblastoma treated with an autologous tumor vaccine

**DOI:** 10.1007/s11060-025-05203-w

**Published:** 2025-09-09

**Authors:** Kazuki Akutagawa, Shunichiro Miki, Erika Yamada, Noriaki Sakamoto, Tsubasa Miyazaki, Narushi Sugii, Alexander Zaboronok, Masahide Matsuda, Eiichi Ishikawa

**Affiliations:** 1 Department of Neurosurgery, Institute of Medicine, University of Tsukuba, 1-1-1 Tennodai, Tsukuba, 305-8575 Ibaraki Prefecture Japan; 2 Department of Diagnostic Pathology, Institute of Medicine, University of Tsukuba, 1- 1-1 Tennodai, Tsukuba, 305-8575 Ibaraki Japan; 3 Cell-Medicine, Inc, Sengen 2-1-6, Tsukuba Science City, 305-0047 Ibaraki Japan

**Correction to: Journal of Neuro-Oncology (2025) 174:709–719**

10.1007/s11060-025-05102-0.

## Introduction

In this article, we analyzed a cohort of 58 patients with newly diagnosed IDH-wildtype glioblastoma (GBM) or IDH-mutant grade 4 astrocytoma who received either standard therapy combined with autologous formalin-fixed tumor vaccine (AFTV) or standard therapy alone. Our findings indicated that negative immunostaining for PIK3R2, as well as negative p53 expression, was associated with prolonged survival in patients treated with AFTV for GBM.

In this article, several errors were identified within the reported data. Firstly, inaccuracies in Table 1 were noted regarding the Karnofsky Performance Status (KPS) and median overall survival (OS) values. These errors were due to left–right labeling mistakes, inclusion of non-preoperative KPS values, and incorrect statistical processing of the median OS. Specifically, the original median OS was calculated as a simple median of all values, whereas the corrected value represents the proper median OS derived from survival curves. Additionally, a statistical error was identified in the gender analysis, where a one-tailed test was used instead of the appropriate two-tailed test.

It is important to emphasize that, aside from two rows in Table 1, all other statistical analyses are accurate. These corrections do not alter the primary findings, discussion, or conclusions of the original article, particularly regarding the prognostic significance of p53 and PIK3R2 expression in patients receiving AFTV therapy.

## Article content

In the Results section,

“In the AFTV group, the mean age was 59.1 years, and 14 patients (48.3%) were female. The median KPS at the time of surgery was 90, and the mOS was 30 months. There were significantly more female patients in the AFTV group compared to the control group (p < 0.05), but no significant differences were observed in other baseline characteristics (Table 1).”

should read as follows:

“In the AFTV group, the mean age was 59.1 years, and 14 patients (48.3%) were female. The median KPS at the time of surgery was 80, and the mOS was 32.6 months. There were more female patients in the AFTV group compared to the control group (*p* = 0.1), but this was not statistically significant (Table 1).”

The below copied upper rows of Table 1 should have the corrected cell entries as shown in the Revised Table 1 below.

**Table:** 

Characteristic	AFTV group (*N* = 29)	Control group (*N* = 29)	Total (*N* = 58)	*P*-value
Age (years, mean [range])	59.1 (39–74)	55.3 (14–85)	57.2 (14–85)	> 0.1
Sex (female, n [%])	14 (48.3)	7 (24.1)	21 (36.2)	0.050
Preoperative KPS (median [range])	90 (40–100)	80 (50–100)	80 (40–100)	> 0.1
Overall survival (months, median [range])	30.0 (6.9-118.2)	29.5 (2.8–82.2)	29.8 (2.8-118.2)	> 0.1

Revised Table 1. Patient characteristics.

**Table:** 

Characteristic	AFTV group (*N* = 29)	Control group (*N* = 29)	Total (*N* = 58)	*P*-value
Age (years, mean [range])	59.1 (39–74)	55.3 (14–85)	57.2 (14–85)	> 0.1
Sex (female, n [%])	14 (48.3)	7 (24.1)	21 (36.2)	**0.100**
Preoperative KPS (median [range])	**80 (50**–100)	**90** (50–100)	80 (**50**–100)	> 0.1
Overall survival (months, median [range])	**32.6** (6.9-118.2)	**31.1** (2.8–82.2)	**31.1** (2.8-118.2)	> 0.1

In the figure legend of Fig. 1, “Fig. 1 PI3R2 staining” should read “Fig. 1 PIK3R2 staining”.

The original article has been corrected.

