# Supplementary material for: PIK3R2 immunostaining status predicts prognosis in patients with newly diagnosed glioblastoma treated with an autologous tumor vaccine
Source: J Neurooncol. 2025 Jun 12;174(3):709–19. doi: 10.1007/s11060-025-05102-0 (PMC12263475; doi:10.1007/s11060-025-05102-0)

**PIK3R2 and p53 immunostaining status predicts prognosis in patients with newly diagnosed glioblastoma treated with an autologous tumor vaccine.**

**Supplementary Table and Figures**

**Supplementary Table 1.** Candidate genes associated with prognosis in AFTV-treated patients, identified through panel testing, excluding genes correlated with p53 mutation status.

| Genes | Correlation with TP53 mutation status | Correlation with prognosis in the control group | Correlation with prognosis in the AFTV group |
| --- | --- | --- | --- |
| BZRAP1-AS1, RNF43 | 0.365 | 0.754 | 0.014 |
| DNM2 | 0.212 | 0.909 | 0.042 |
| TNFAIP3 | 0.561 | 0.898 | 0.069 |
| MET | 0.260 | 0.959 | 0.069 |
| MTOR | 0.453 | 0.659 | 0.077 |
| CBLC | 0.453 | 0.332 | 0.078 |
| PIK3R2 | 0.590 | 0.532 | 0.079 |
| EZH2 | 0.345 | 0.179 | 0.084 |
| EP300, RP1-85F18.6, RP1-85F18.5 | 0.453 | 0.419 | 0.093 |
| CSF3R | 0.722 | 0.089 | 0.107 |

**Supplementary Table 2.** Median overall survival (mOS (months)) of each subgroup treated with standard therapy alone (Control group) or standard therapy plus AFTV (AFTV group) for GBM/Astro (Upper) and GBM only (Lower).

| GBM/Astro group | mOS (months) in Control group | | mOS (months) in AFTV group | |
| --- | --- | --- | --- | --- |
| p53 status | p53 negative | p53 positive | p53 negative | p53 positive |
| **PIK3R2 negative** | **31.1** | **38.7** | **65.6** | **7.4** |
| **PIK3R2 positive** | **35.8** | **30.3** | **38.7** | **21.2** |
| PD1 negative | 42.2 | 30 | 53.1 | 16.5 |
| PD1 positive | 22.7 | 9.8 | 38.7 | 20.2 |
| CD8 negative | 26.8 | 16.7 | 16.8 | 16.5 |
| CD8 positive | 39.2 | 36.9 | 118.2 | 20.2 |
| CD3 negative | 21.4 | 16.7 | 49.8 | 16.5 |
| CD3 positive | 39.2 | 36.9 | 65.6 | 20.2 |
| CD163 negative | 21.4 | 30 | 49.8 | 24.5 |
| CD163 positive | 21.4 | 9.8 | 118.2 | 8.5 |
| PDL1 negative | 22.7 | 30 | 49.8 | 21.2 |
| PDL1 positive | 39.2 | 15.2 | 65.6 | 16.5 |
| CD20 negative | 26.8 | 23.5 | 118.2 | 20.2 |
| CD20 positive | 39.2 | 36.9 | 34.4 | 6.9 |

| GBM only | mOS (months) in Control group | | mOS (months) in AFTV group | |
| --- | --- | --- | --- | --- |
| p53 status | p53 negative | p53 positive | p53 negative | p53 positive |
| **PIK3R2 negative** | **31.1** | **8.9** | **65.6** | **7.4** |
| **PIK3R2 positive** | **35.8** | **30.0** | **38.7** | **20.2** |
| PD1 negative | 42.2 | 23.5 | 53.1 | 16.5 |
| PD1 positive | 22.7 | 9.8 | 38.7 | 15.5 |
| CD8 negative | 21.4 | 16.7 | 16.8 | 15.5 |
| CD8 positive | 39.2 | 23.5 | 118.2 | 20.2 |
| CD3 negative | 21.4 | 16.7 | 49.8 | 12.1 |
| CD3 positive | 35.8 | 23.5 | 65.6 | 20.2 |
| CD163 negative | 35.8 | 23.5 | 38.7 | 20.2 |
| CD163 positive | 21.4 | 8.9 | 118.2 | 8.5 |
| PDL1 negative | 22.7 | 23.5 | 49.8 | 21.2 |
| PDL1 positive | 39.2 | 9.8 | 65.6 | 8.5 |
| CD20 negative | 21.4 | 23.5 | 118.2 | 16.5 |
| CD20 positive | 39.2 | 9.9 | 34.4 | 6.9 |

**Supplementary Figure 1.** GlioVis analysis using HG-U133A gene expression data shows no correlation between PIK3R2 expression levels and patient prognosis in a single-gene analysis. The settings in the GlioVis Explore were as follows: the Dataset was set to *Adult TCGA_GBM*, the Platform was set to *HG-U133A*, the Histology to *GBM*, and all options for Subtype, Gender, IDH status, Recurrence, and MGMT status were set to *All*. The expression cutoff used was the *median*.


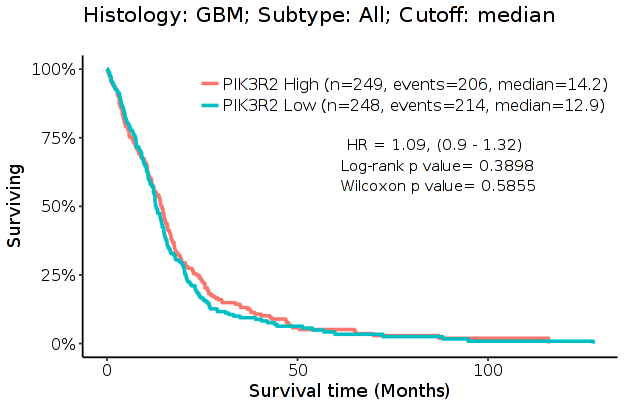


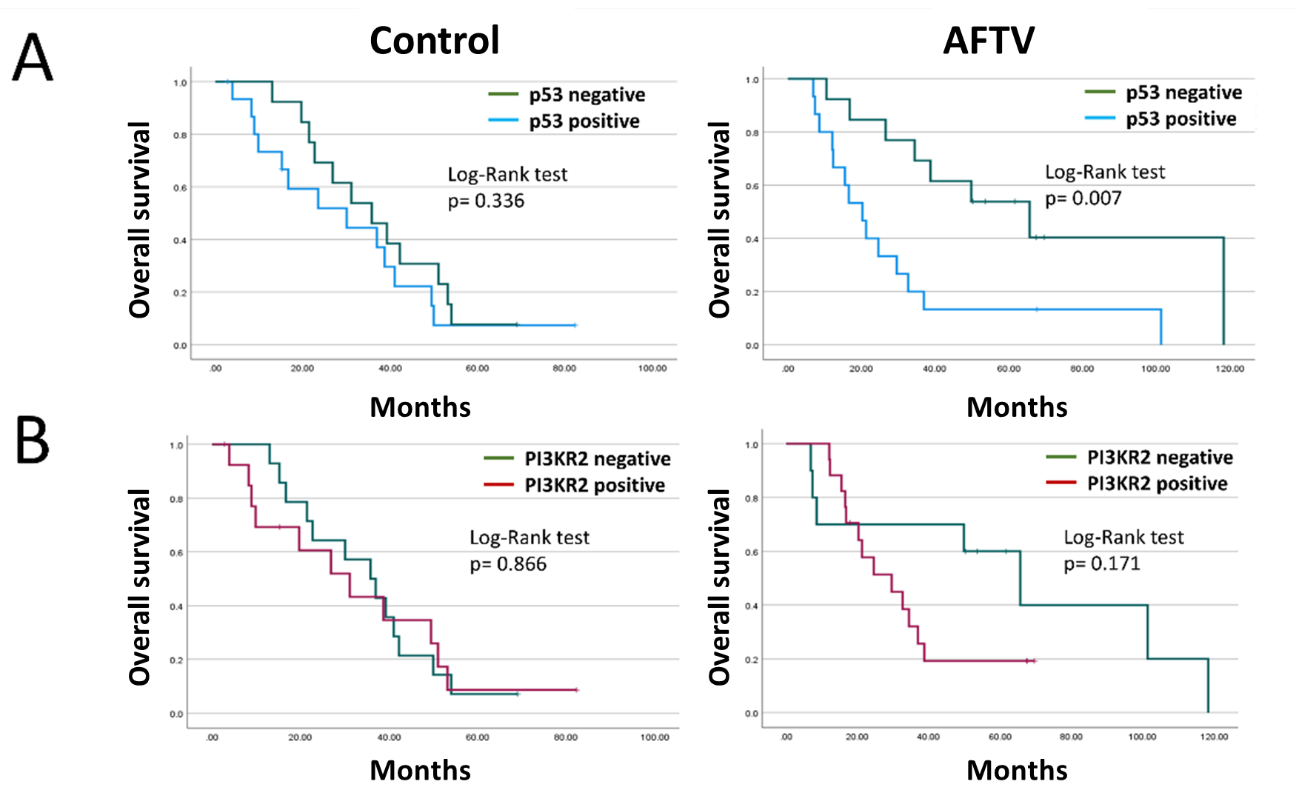
**Supplementary Figure 2.** Comparison of overall survival (OS) between the AFTV and control groups based on p53 and PIK3R2 mutation status. (**A**) In the control group, OS did not significantly differ based on p53 status. However, in the AFTV group, OS was significantly longer in p53-negative patients, suggesting that p53-negative status may predict AFTV efficacy. (**B**) In the control group, OS did not differ based on PIK3R2 status. In the AFTV group, although the difference was not statistically significant, the PIK3R2-negative subgroup showed a trend toward longer survival.

**Supplementary Figure 3.** Comparison of overall survival (OS) among four groups based on the combination of p53 and PIK3R2 mutation status. In the control group, there was no significant difference in OS among the four groups. However, in the AFTV group, the p53-negative/PIK3R2-negative subgroup (indicated by the orange line) had a significantly longer median OS of 65.6 months.


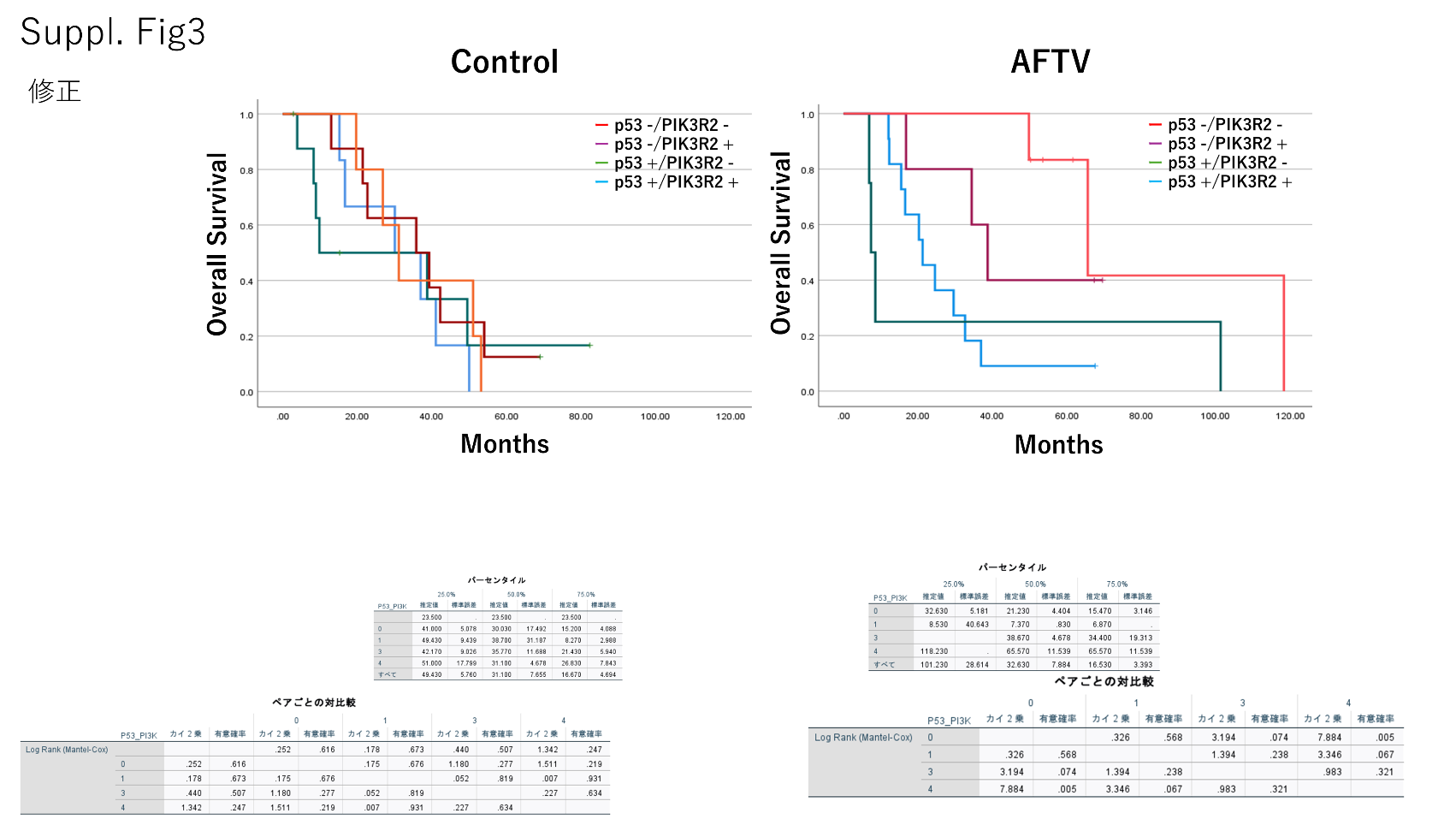

Supplement: Supplementary file 1 — Supplementary Material 1 [file 11060_2025_5102_MOESM1_ESM.docx]
